# Emergency Department Point-of-Care Ultrasound Diagnosis of a Large Bowel Obstruction Due to Metastatic Rectal Cancer: A Case Report

**DOI:** 10.7759/cureus.28817

**Published:** 2022-09-05

**Authors:** Derrick Huang, Mortatha Al-Bassam, Leoh N Leon, Latha Ganti

**Affiliations:** 1 Emergency Medicine, Ocala Regional Medical Center, Ocala, USA; 2 Emergency Medicine, Osceola Regional Medical Center, Orlando, USA; 3 Emergency Medicine, HCA Florida Ocala Hospital, Ocala, USA; 4 Emergency Medicine, Envision Physician Services, Plantation, USA; 5 Emergency Medicine, University of Central Florida College of Medicine, Orlando, USA

**Keywords:** abdominal pain, ultrasound, acoustic enhancement, colorectal cancer, rectal cancer, mechanical large bowel obstruction, point-of-care-ultrasound, emergency medicine

## Abstract

Large bowel obstruction (LBO) is a life-threatening condition seen most often in the geriatric population. LBO can present with nonspecific abdominal pain that can overlap with other pathologies, such as abdominal infection, acute aortic disease, intestinal perforation, and atypical acute coronary syndrome in the geriatric population. Delays in diagnosis of colonic obstruction result in significant mortality due to complications involving bowel necrosis, perforation, and sepsis. In the emergency department (ED), abdominal point-of-care ultrasound (POCUS) can diagnose LBO and facilitate the assessment of the wide differential inherent to elderly abdominal pain. The authors report a rare ED application of abdominal POCUS to facilitate rapid diagnosis of an LBO secondary to rectal cancer.

## Introduction

Large bowel obstruction (LBO) in the emergency department (ED) is a life-threatening pathology encountered most commonly in the geriatric population that accounts for about 2-4% of all surgical admissions [1-2]. The most common causes of LBO in adults are colorectal cancer followed by diverticulitis and volvulus, which together account for about 80-85% of all LBO cases [2]. LBOs are 4-5 times less common than small bowel obstructions (SBOs) and constitute approximately 10-15% of all intestinal obstructions [1-2]. Upon progression of the bowel obstruction, intraluminal pressure increases and colonic distension worsens proximal to the transition point, clinically manifesting as abdominal pain, constipation or obstipation, and abdominal distention [2]. Vomiting may also be present if the patient has ileocecal valve incompetency [2]. Without timely diagnosis and treatment, this pathology may ultimately result in bowel ischemia and infarction leading to perforation with a mortality rate as high as 15% if emergency surgery is required in cases of colorectal malignancies [1,3].

In the ED, the primary focus of a geriatric patient presenting with undifferentiated abdominal pain is expeditious diagnosis and stabilization of life-threatening pathologies. In addition to an often-nonspecific examination and unreliable vital signs, geriatric abdominal pain is complicated by a wide differential that includes dangerous etiologies, such as atypical acute coronary syndrome, gastrointestinal perforation, aortic pathology, peritonitis, and bowel obstruction [4-5]. In additional to colorectal cancer, which constitutes 60% of LBO cases, extracolonic neoplasms contribute to 10% of cases, typically involving extrinsic bowel compression from peritoneal malignancy, direct invasion, or lymphatic or hematogenous metastasis [2,6]. Furthermore, volvulus and diverticulitis, when complicated with inflammatory bowel adhesions, pericolic fibrosis, and large abscesses, are responsible for approximately 10-15% and 10% of cases, respectively [2]. Providers should also be concerned of LBO mimics, including non-mechanical pseudo-obstruction, or Ogilvie's syndrome, and toxic megacolon related to inflammatory bowel disease [2]. ED point-of-care ultrasound (POCUS) has an established role in resuscitation and the assessment of various abdominal pathologies, such as aortic disease and intraperitoneal free fluid, and can expedite the evaluation of the wide differential inherent in geriatric abdominal pain [7-8]. Although computed tomography (CT) imaging is the definitive imaging modality in the diagnosis of LBO, we present a novel case of rectal cancer presenting with an LBO that was rapidly diagnosed with abdominal POCUS [2].

## Case presentation

An 82-year-old male with a history of hypertension, prior smoking, coronary artery disease, and remote history of prostate cancer status post chemotherapy and radiation presented to the ED with progressively worsening abdominal pain. He stated that the pain was nonspecific in location, without radiation, and present for several weeks before acutely worsening in the last four days. He reported nausea without vomiting and stated that he was obstipated over the last four days. He reported previously having episodes of diarrhea and some recent weight loss. He denied rectal bleeding, fevers, chest pain, dyspnea, and genitourinary symptoms. He was unsure when he had his last colonoscopy but stated that it was a long time ago. He denied the use of beta blockers and anticoagulants. 

**Figure 1:**
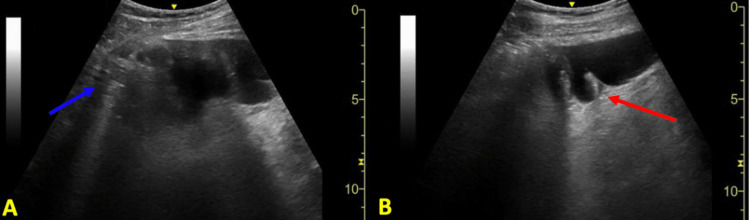
Sonographic image of the left lower quadrant Bedside point-of-care ultrasound (POCUS) image utilizing a low-frequency curvilinear probe at the left lower quadrant showing proximal (A) and distal (B) views of the distal descending colon. There is colonic dilation measuring maximally at about 5 cm. There are thickened plicae circulares measuring approximately 3 mm (red arrows) and abdominal A lines (blue arrow).

**Figure 2:**
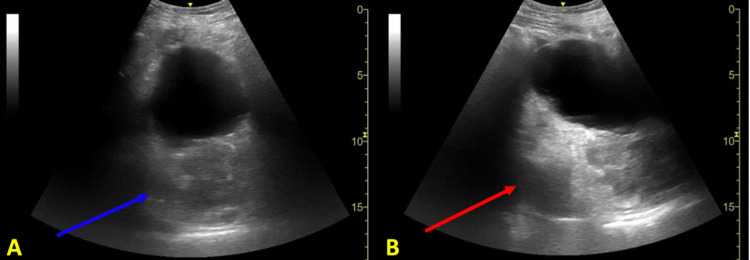
Sonographic image of the suprapubic region Bedside POCUS image utilizing a low-frequency curvilinear probe at the suprapubic region with transverse (A) and sagittal (B) views showing the bladder with posterior acoustic enhancement. There is a poorly defined, heterogenous mass lesion deep to the bladder in the rectosigmoid region measuring approximately 7 cm (blue arrow). There is evidence of colonic dilation proximal to the mass lesion (red arrow).

On his initial vital signs, he had a heart rate of 107 beats per minute, respiratory rate of 16 breaths per minute, and blood pressure of 212/103 mmHg. He was afebrile with a normal oxygen saturation on room air. He appeared frail and distressed. His abdomen was softly distended with nonperitoneal tenderness to palpation. His labs were remarkable for a lactic acid of 3.3 mmol/L. White blood cell counts, renal and hepatic function tests, lipase, and electrolytes were unremarkable. His troponin and electrocardiogram were unremarkable. Intravenous fluids were administered. POCUS was performed on the patient and was remarkable for findings of a bowel obstructing rectal mass (Figures 1-2).

**Figure 3:**
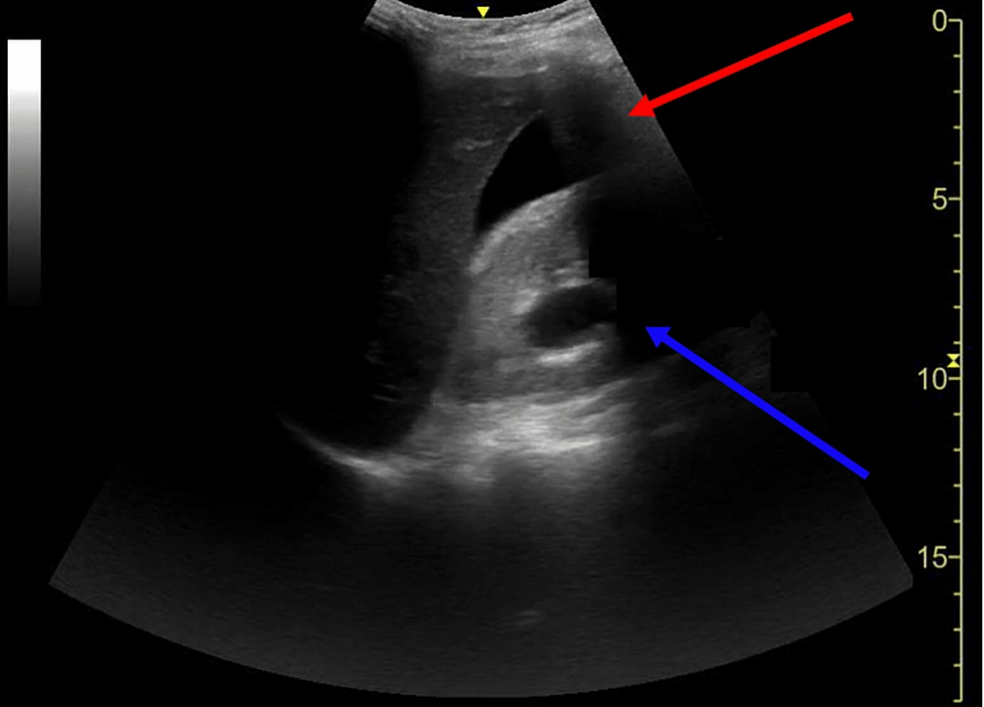
Sonographic image of the right upper quadrant Bedside POCUS image utilizing a low-frequency curvilinear probe at the right upper quadrant showing intraperitoneal free fluid in Morrison’s pouch in the setting of a newly diagnosed rectal mass lesion (red arrow). There is evidence of hydronephrosis in the right kidney (blue arrow).

Intra-abdominal free fluid was also identified in the setting of possible metastatic cancer (Figure 3).

**Figure 4:**
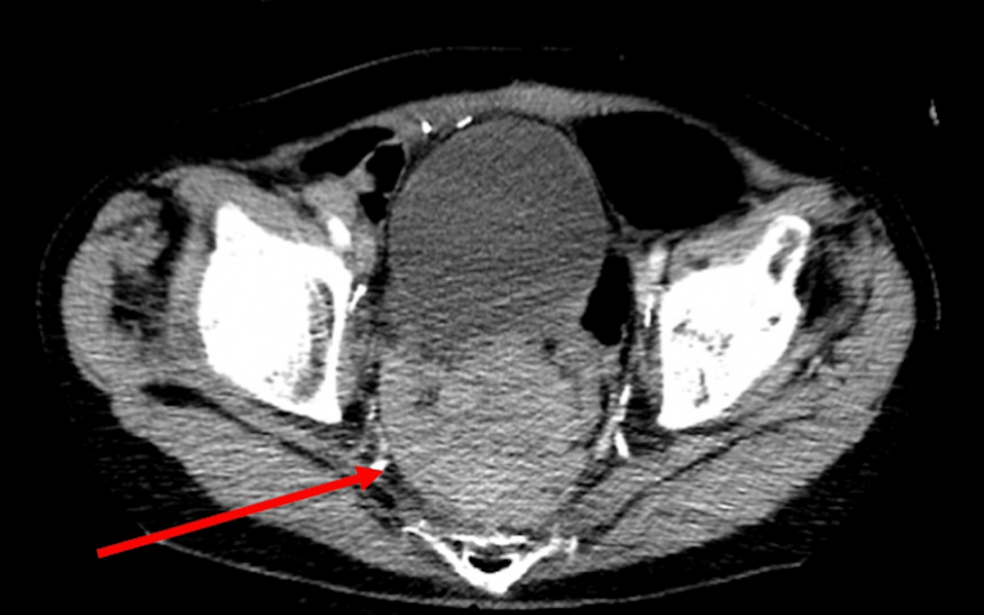
Contrast computed tomography (CT) of the pelvis showing a rectal mass Transverse view of the pelvis showing wall thickening and an enhancing mass lesion of the rectosigmoid colon obliterating tissue planes in the low pelvis and encasing the rectum measuring about 8 cm (red arrow).

**Figure 5:**
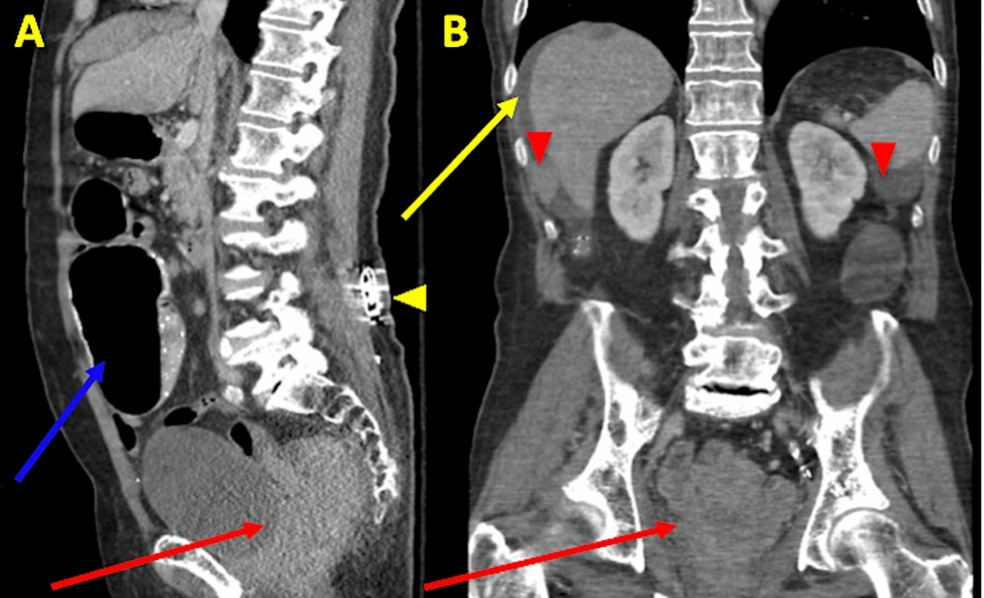
Contrast CT imaging of the abdomen and pelvis showing a rectal mass and evidence of metastasis Sagittal (A) and coronal (B) images of the abdomen and pelvis showing wall thickening of the rectum with a large mass lesion obliterating the surrounding tissue planes along the rectosigmoid colon and encasing the rectum measuring approximately 5.6 cm x 7.6 cm (red arrows). The colon is dilated suggesting distal obstruction (blue arrow). There is a moderate amount of perihepatic fluid (yellow arrow) and multiple nodules throughout the abdomen concerning metastatic disease (red arrow tips). There is a sacral nerve stimulator (yellow arrow tip).

Findings were confirmed on CT of the abdomen and pelvis with contrast, which was also significant for numerous enlarged retroperitoneal and periaortic lymph nodes, a moderate amount of perihepatic fluid, and multiple masses on the liver, diaphragm, and the peritoneum (Figures 4-5).

After evaluation by the surgical team, the patient was admitted for medical management. A sigmoidoscopy with biopsy was performed and found a large obstructive lesion involving the entire rectum and rectosigmoid area. Ultimately, the patient was diagnosed with Stage 4 rectal cancer. Given the deconditioned state of the patient, he was not a candidate for chemotherapy. He deferred palliative colostomy and was ultimately scheduled for rectal stent placement and placement into palliative care.

## Discussion

We present a case of metastatic rectal cancer presenting with an LBO and diagnosed with abdominal POCUS using both suprapubic and standard abdominal quadrant views. Obstruction complicates up to 29% of colorectal cancers, accounting for about 80% of emergency presentations of colorectal cancer [2,9]. Over 75% of obstructing colorectal cancers occur distal to the splenic flexure with the most common site at the sigmoid colon due to its relatively narrow diameter, whereas rectal obstruction comprises <10% of cases of LBO [2,6]. In the geriatric population, undifferentiated nonspecific abdominal pain can be difficult to assess [10]. Although LBOs have classic findings of abdominal pain, constipation or obstipation, and distention, diagnosis can be complicated by a varied presentation based on the progression of obstruction. In addition to an often-unreliable clinical exam in geriatric patients, symptoms of LBO secondary to cancer initially manifest insidiously with prolonged mild, crampy pain before increasing in intensity with worsening bowel distension [4-5,10]. Paradoxical decreases in pain may accompany the reduced bowel motility associated with worsening distension [10]. The difficulty of the clinical examination is underscored by about one-third of patients suspected of having a mechanical obstruction on the exam and abdominal radiography ultimately having no obstruction [11]. Conversely, about 20% of patients suspected of having a colonic pseudo-obstruction may ultimately have a mechanical etiology [11]. Given these difficulties, emergency providers should supplement their clinical exam with abdominal POCUS to facilitate the better assessment of patients presenting with symptoms of abdominal obstruction.

The utilization of abdominal POCUS has been well-established in ED resuscitation and abdominal pain [7-8]. Additionally, in several studies POCUS has been clinically impactful in expediting diagnosis and risk stratification in patients with SBO and diverticulitis in the correct clinical setting. In SBO, POCUS may have sensitivity and specificity values as high as 92.4% and 96.6%, respectively, with sonographic findings of dilated, fluid-filled bowel loops >2.5 cm and increased peristalsis [12]. For diverticulitis, POCUS may have sensitivity and specificity values of up to 92% and 97%, respectively, for uncomplicated diverticulitis with sonographic findings of bowel wall thickening >5 mm around a diverticulum, pericolonic fat enhancement, and sonographic tenderness to palpation [13-14]. Although the use of POCUS for LBO has not been as extensively studied when compared to SBOs, an interdisciplinary group of experts has suggested that ultrasound may be able to diagnose LBO with diagnostic criteria involving visualization of a large intestine >4.5 cm in diameter, abdominal A-lines, abnormal bowel peristalsis, and thickened plicae circulares >2 mm [15-16]. Sonographic visualization of a transition point between dilated and collapsed bowel can also localize the occlusion. In our case, POCUS visualized a rectal mass deep to the bladder with resultant bowel distension (Figures 1 and 2). Intra-abdominal free fluid and hydronephrosis were also identified on the right upper quadrant abdominal view (Figure 3). In clinical correlation with a new rectal mass, nonperitoneal abdominal tenderness, and stable vital signs, the visualized free-fluid was presumed as ascites secondary to metastatic cancer whereas the hydronephrosis was likely related to metastatic ureteral compression. On the left lower quadrant abdominal view, visualization of 5 cm of colonic dilation, 3 mm plicae circulares, and abdominal A lines was consistent with an LBO (Figure 1). In addition, ultrasound can also assess for free air, which may be sonographically visualized as echogenic free fluid, reverberation artifact, and a shifting enhanced peritoneal stripe sign, as well as intraperitoneal free fluid with sensitivity and specificity values of 74% and 96%, respectively [15,17-18]. Although POCUS has tremendous use in bedside evaluation and when CT imaging is unavailable, CT remains the definitive imaging modality in LBOs for detecting the etiologies and complications of obstruction [2].

Management of LBO in the ED centers on stabilization and risk stratification. Symptomatic control and supportive treatment with antiemetics, bowel rest, as-needed nasogastric decompression, and intravenous hydration should be administered with consideration for avoiding iatrogenic fluid overload in patients with renal or cardiac insufficiency [1]. Appropriate broad-spectrum antibiotics covering abdominal flora should also be administered in the correct clinical setting. Importantly, the provider must assess for complications of LBO that will alter management. In the setting of colorectal cancer, complications may include infarction or bowel ischemia, perforation, and peritonitis [1-2]. Definitive treatment of LBOs is determined by the surgical team and based on etiology, patient comorbidities, severity, and presence of complications [19]. Conservative treatment in the case of an uncomplicated LBO without symptoms and signs of hypovolemic or septic shock is preferred over invasive surgical intervention, as partial or low-grade obstructions often resolve with nasogastric tube decompression and supportive measures [1]. Generally, given poor outcomes after emergency surgical intervention and a high rate of postoperative complications in older patients with LBOs, surgical intervention is only indicated when conservative treatment fails or in the setting of a complicated LBO. Such intervention is also time-sensitive - typically recommended 24-48 hours after hospital admission [1-2]. In the setting of colorectal cancer, surgery is location dependent and generally considered only after endoscopic dilatation, endoscopic colonic stent placement, and clinical stabilization [1,19]. A delayed diagnosis of LBO correlates with poorer outcomes, underscoring the importance of rapid diagnosis and stabilization in the ED [1]. 

## Conclusions

Delays in diagnosis of colonic obstruction can result in significant mortality and morbidity due to complications involving bowel necrosis, perforation, and sepsis. In the ED, POCUS possesses potential utility in both expeditiously diagnosing LBOs as well as assessing for complications of obstruction.
